# Study on Light Interception and Biomass Production of Different Cotton Cultivars

**DOI:** 10.1371/journal.pone.0156335

**Published:** 2016-05-26

**Authors:** Zhigang Bai, Shuchun Mao, Yingchun Han, Lu Feng, Guoping Wang, Beifang Yang, Xiaoyu Zhi, Zhengyi Fan, Yaping Lei, Wenli Du, Yabing Li

**Affiliations:** Institute of Cotton Research of Chinese Academy of Agricultural Sciences/State Key Laboratory of Cotton Biology, Anyang, 455000, Henan, China; Nanjing Agricultural University, CHINA

## Abstract

Identifying the characteristics of light interception and utilization is of great significance for improving the potential photosynthetic activity of plants. The present research investigates the differences in absorbing and converting photosynthetically active radiation (PAR) among various cotton cultivars. Field experiments were conducted in 2012, 2013 and 2014 in Anyang, Henan, China. Ten cultivars with different maturity and plant architectures were planted at a density of 60,000 plants ha^-1^ in randomized blocks, with three replicates. The spatial distribution of light in canopy was measured and quantified with a geo-statistical method, according to which the cumulative amount of intercepted radiation was calculated by Simpson 3/8 rules. Finally, light interception was analyzed in association with the biomass accumulation of different cultivars. The key results were: (1) late-maturing varieties with an incompact plant architecture captured more solar radiation throughout the whole growth period than middle varieties with columnar architecture and even more than early varieties with compact architecture, and they produced more biomass; (2) the highest PAR interception ratio and the maximum biomass accumulation rate occurred during the blossoming and boll-forming stage, when leaf area index (LAI) reached its peak; (3) the distribution within the canopy presented a significant spatial heterogeneity, and at late growing stage, the PAR was mainly intercepted by upper canopies in incompact-type plant communities, but was more homogeneous in columnar-type plants; however, the majority of radiation was transmitted through the canopy in compact-type colonies; (4) there was not a consistent variation relationship between the cumulative intercepted PAR (iPAR) and biomass among these cultivars over the three years of the study. Based on these results, we attempted to clarify the distinction in light spatial distribution within different canopies and the patterns of PAR interception in diverse cotton cultivars with different hereditary characters, thereby providing a significant basis for researchers to select cultivars with appropriate growth period and optimal plant architecture for improvement of light interception and utilization.

## Introduction

Solar radiation provides a free energy source for plant growth [1], but only the photosynthetically active part of the spectrum (400–700 nm) can directly drive photosynthesis. It is defined as Photosynthetically Active Radiation (PAR) [1–2]. The ability to absorb and convert PAR immediately reflects crop biomass productivity, which is the foundation of crop economic yield [3].

Louarn G. expressed that crop biomass production was attributed to the following two factors: amount of PAR intercepted by the canopy and efficiency of converting the captured radiation into biomass, i.e., Radiation Use Efficiency (RUE) [4]. Further studies indicate that the green leaf area, its duration of exposure, and the canopy structure mainly govern the first attribute of PAR interception; RUE is largely controlled by plant’s net-photosynthetic capacity, as well as by canopy structure to some extent [5].

Numerous studies have demonstrated that canopy structure significantly influences plant radiation interception through regulating the light distribution within the canopy [5–10]. Consequently, to fully understand the underlying processes of radiation capture and energy fixation into biomass, it is crucial to deeply explore the detailed and precise characteristics of light distribution in the plant canopy [11]. The present work was also focused on this concern.

Since Monsi and Saeki introduced the application of Beer’s Law to estimate the light extinction and interception in a plant canopy [12], many researchers have attempted to clarify the relationship between light distribution and interception by using numerous methods. The classical approach for investigating and estimating light distribution within the crop canopy was to use mathematical functions based on the Beer-Lambert law with parameters such as extinction coefficient (k), LAI, and mean leaf tilt angle [13].

While in recent years, benefitting from the rapid development of modern computer technology, the functional-structural plant models (FSPMs), also known as the three-dimensional (3-D) virtual plant models, have been a versatile and effective tool for simulating the interaction between plants and their growth environment, especially in describing light distribution and interception [14]. For example, Herve Rey introduced a 3-D virtual sunflower model, which was coupled with the AMAPsim architectural and MMP radiative calculation software, to simulate the amount of radiation intercepted over the sunflower’s growth period [15]. H. Sinoquet proposed a method for constructing 3-D plant models using 3-D digitizing and image processing to characterize the light environment in canopies [10]. Moreover, Ma et al. also built a model for quickly computing the light distribution within canopies based on a virtual camera [16].

However, these functions and models failed to be completely consistent with the actual situation because of a common defect of considering the plant canopy as a homogeneous medium with random distribution of leaf orientation [17]. As the canopy is irregular and discontinuous, spatial heterogeneity effects should be taken into consideration when studying light distribution and interception characteristics in plant canopies [18]. Difficulties remain when measuring the light distribution in canopies, namely, how to precisely quantify the characteristics of light spatial distribution in canopies. Here, a geo-statistical grid sampling method based on spatial statistics was used for exploring PAR variation and distribution in a heterogeneous canopy [19].

For cotton (*Gossypium hirsutum* L.), as a heliophile, adequate light interception is essential to its growth [20]. Understanding the effects of plant architecture on radiation interception by cotton can be very useful for optimizing canopy architecture to obtain more solar radiation and then to improve the growth managements for a higher cotton lint yield [21]. However, this is more difficult to accomplish in cotton than in other crops, such as maize, rice or wheat, because cotton is an indeterminate perennial plant cultivated as an annual crop, which leads to a more complicated canopy structure. [22]

To identify and select an optimal cotton cultivar that has a high efficiency for intercepting and converting the solar radiation, as well as to better understand the characteristics of radiation interception and dry matter accumulation of diverse cotton cultivars, we set up the experiments described below. We chose several cotton cultivars of differing plant architecture and maturity for the purpose of building diverse canopy structures. Spatial distribution of light in the canopy was accurately measured, and the cumulative amount of light interception and biomass production were calculated. Based on this work, we aimed to identify the characteristics of light distribution in different canopy structures and how plant architecture affects PAR interception and transformation, as well as to contribute to optimizing an idealized cotton shape which has high productivity.

## Materials and Methods

### 1. Experimental design

This study was conducted in 2012–2014 at the experimental field of the Cotton Research Institute of the Chinese Academy of Agricultural Sciences in Anyang, Henan, China (longitude 36°06′N, latitude114°21′E). The experimental plot was a cotton field with long-term continuous cropping, and the soil at the experimental field was a medium loam soil with a total N 0.66 g·kg^-1^, P 0.01 g·kg^-1^ and K 0.11 g·kg^-1^. During the cotton development stage (from April to October), the average temperature was 22.27°C in 2012, 21.96°C in 2013 and 22.03°C in 2014. At the same time, total rainfall was 437.2 mm, 428.9 mm and 450.5 mm in 2012, 2013, and 2014, respectively.

The experimental design was a randomized complete block with ten different selected cotton cultivars (nine cultivars in 2012), and was replicated three times with a plot size of 8 m×8 m. Among these cultivars, Ji958, Ji228, 3799 and 6913 have longer growth periods and tend to extend horizontal fruiting branches. However, cultivars such as CRI60, CRI79, and Lu28 tend towards shorter branches, smaller leaves and a shorter growth period. In contrast, branches of T-0, 113 and 915 are shorter and have serried fruit nodes, and all of them are early-maturing varieties. Each plot was composed of ten rows, 0.8 m apart, and the crops were oriented north-south. The field was fully plowed and irrigated in early spring before sowing. Sowing dates were 22 April, 17 April and 30 April in 2012, 2013 and2014, respectively, and plants were manually thinned out to the desired density of 60,000 plants ha^-1^ at the two-leaf stage. All plots received fertilizer at 225.0 kg·ha^-1^ N, 150.0 kg·ha^-1^ P, and 225.0 kg·ha^-1^ K, as well as a total volume of approximately 40 m^3^ water by flooding the furrows to ensure that cotton growth was free of nutrient and water restrictions over the entire cropping season. Herbicides and pesticides were adequately used to control the weeds, insects and diseases aimed at avoiding the stress of adverse conditions.

### 2. Data collection

#### 2.1 PAR interception and transmission in the canopy

In our experiment, the intensity of transmitted PAR (TPAR) and reflected PAR (RPAR) in different canopy layers were measured by the spatial grid sampling method using a portable 1.0 m line light quantum sensor (LI-191SA,LI-COR,Lincoln,NE,USA) and datalogger (LI-1400, LI-COR). Measurements were taken every ten days in the same sample area in each plot during the crop season between the four-stage and maturity in each year and were performed at 10:00 am under clear skies. Between two rows, 80 cm was segmented into four horizontal sections of 20 cm each. Thus, we had 5 measuring positions at 0 cm, 20 cm, 40 cm, 60 cm and 80 cm from a west row to the adjacent east row. The canopy was also divided into several thin vertical layers of per 20 cm from the bottom of the canopy, with the number of layers varying according to the plant height. At every point, the TPAR and RPAR were measured. The process was repeated up to the layer above the canopy. This measurement provided a comprehensive set of spatial data for light intensities within the canopy. Moreover, a second line quantum analyzer was placed 20 cm above the crop canopy and was set to automatically record the incident PAR (IPAR) at 5 second intervals.

Then, a fraction of transmitted PAR (tPAR), reflected PAR (rPAR) and intercepted PAR (iPAR) were calculated using the following equations from Tang[23]:

$$\text{t}\;PAR=\frac{TPAR}{I\;PAR}$$ (1)

$$\text{r}\;PAR=\frac{RPAR}{I\;PAR}$$ (2)

$$\text{i}\;PAR=\frac{I\;PAR-TPAR-RPAR}{I\;PAR}=1-t\;PAR-rPAR$$ (3)

However, from the study of ZHI, we learn that the rPAR of cotton in a density of 60,000 plants ha^-1^ was under 5% during the entire cropping period [18]. Therefore, in this paper we ignore the influence of the rPAR. The simplified Eq 3 is as follows:

iPAR=1−tPAR (4)

#### 2.2 Estimation of PAR distribution in the canopy

In other positions within the canopy, iPAR and tPAR values were calculated by spatial interpolation with the following equation from Li [24]:

$$Z(X_{0})=\sum_{i=1}^{n}\lambda_{\text{i}}Z(X_{\text{i}})$$ (5)

where Z(X_0_) is the measured PAR values, λ_i_ is the coefficient of the sample, and the unbiased condition ∑*λi* = 1 was employed.

Furthermore, based on the minimum variance, the Kriging equation was stated as follows:

$$\sum_{i=1}^{n}\lambda_{i}\gamma (x_{i},x_{j})+\varphi =\gamma (x_{i},x_{0})\;\text{i}=1,\;2,3,\ldots \;,n$$ (6)

Here, γ(x_i_, x_j_) is the measured value of the variation function, φ is the Lagrangian, γ(x_i_,x_0_) is the measured and calculated PAR, and x_0_ is the estimated value of the calculated point as computed by the unbiased estimate.

#### 2.3 Calculation of accumulated tPAR within the whole canopy

The accumulated tPAR within the whole canopy of the plant group was calculated by the Simpson 3/8 rules based on the Surfer software V12 (Golden Software Inc., USA) [18]. The equation was as follows:

$$\begin{array}{l} A_{i}=\frac{3\Delta x}{8}(G_{i,1}+3G_{i,2}+3_{Gi,3}+2_{Gi,4}+\ldots +2G_{\text{i},ncol\;-\;1}+G_{i,ncol});\\ Volume\approx \frac{3\Delta y}{8}(A_{1}+3A_{2}+3A_{3}+2A_{4}+\ldots +2A_{ncol\;-\;1}+A_{ncol}) \end{array}$$ (7)

where the coefficient vector is [*5*, *3*, *3*, *2*, *…*, *3*, *3*, *2*,*1*], Δx is the vertical distance of the grid, Δy is the horizontal distance; G(i,j) is the grid node number, and volume is the total light volume of a certain cross-sectional area.

#### 2.4 Leaf area index (LAI), biomass accumulation and duration of growth period

The sampling investigation about LAI and biomass was conducted simultaneously with the measurement of the radiation. Two randomly selected plants from each plot were harvested. At least two edge rows were discarded to avoid boundary effects. Leaf area was determined by taking photographs with a scanner (Phantom 9800xl, MiCROTEK, Shanghai, China), and then leaf areas were calculated using Image-Pro Plus software (Media Cybernetics, Inc.)[25]. LAI was computed as the ratio of total green area per unit soil area. After the measurements, plants were dried to a constant dry weight at 80°C for calculating the total dry-matter[26].

Meanwhile, cotton development was surveyed each week, and the dates of reaching every growth stage were recorded. Additionally, the whole duration of cotton development was calculated from sowing to half of the bolls being open (Table 1) [20].

**Table 1:** Date of different growth stages for ten cultivars in 2012, 2013 and 2014.

	Year 2012					Year 2013					Year 2014				
	Seeding Date	Squaring Stage	Flowering Stage	Boll-opening Stage	Duration (days)	Seeding Date	Squaring Stage	Flowering Stage	Boll-opening Stage	Duration (days)	Seeding Date	Squaring Stage	Flowering Stage	Boll-opening Stage	Duration (days)
CRI60	22-Apr	5-Jun	1-Jul	22-Aug	123	17-Apr	6-Jun	8-Jul	28-Aug	134	29-Apr	5-Jun	4-Jul	22-Aug	116
113	22-Apr	1-Jun	28-Jun	19-Aug	120	17-Apr	5-Jun	5-Jul	23-Aug	129	29-Apr	5-Jun	2-Jul	18-Aug	112
Ji228	22-Apr	4-Jun	2-Jul	26-Aug	127	17-Apr	7-Jun	6-Jul	29-Aug	135	29-Apr	7-Jun	5-Jul	25-Aug	119
Ji958	22-Apr	4-Jun	3-Jul	3-Sep	135	17-Apr	7-Jun	7-Jul	30-Aug	136	29-Apr	8-Jun	4-Jul	25-Aug	119
Lu28	22-Apr	3-Jun	29-Jun	23-Aug	124	17-Apr	5-Jun	6-Jul	27-Aug	133	29-Apr	5-Jun	3-Jul	22-Aug	116
915	22-Apr	2-Jun	27-Jun	17-Aug	118	17-Apr	5-Jun	6-Jul	23-Aug	129	29-Apr	4-Jun	1-Jul	19-Aug	113
CRI79	22-Apr	3-Jun	2-Jul	28-Aug	129	17-Apr	6-Jun	7-Jul	29-Aug	135	29-Apr	5-Jun	4-Jul	22-Aug	116
3799	22-Apr	31-May	29-Jun	24-Aug	125	17-Apr	7-Jun	6-Jul	28-Aug	134	29-Apr	5-Jun	5-Jul	24-Aug	118
T-0	22-Apr	28-May	25-Jun	18-Aug	119	17-Apr	31-May	1-Jul	20-Aug	126	29-Apr	1-Jun	27-Jun	15-Aug	109
6913						17-Apr	5-Jun	6-Jul	28-Aug	134	29-Apr	4-Jun	4-Jul	22-Aug	116

## Results

### 1. iPAR variation of different cultivars throughout the growing season

**Fig 1:**
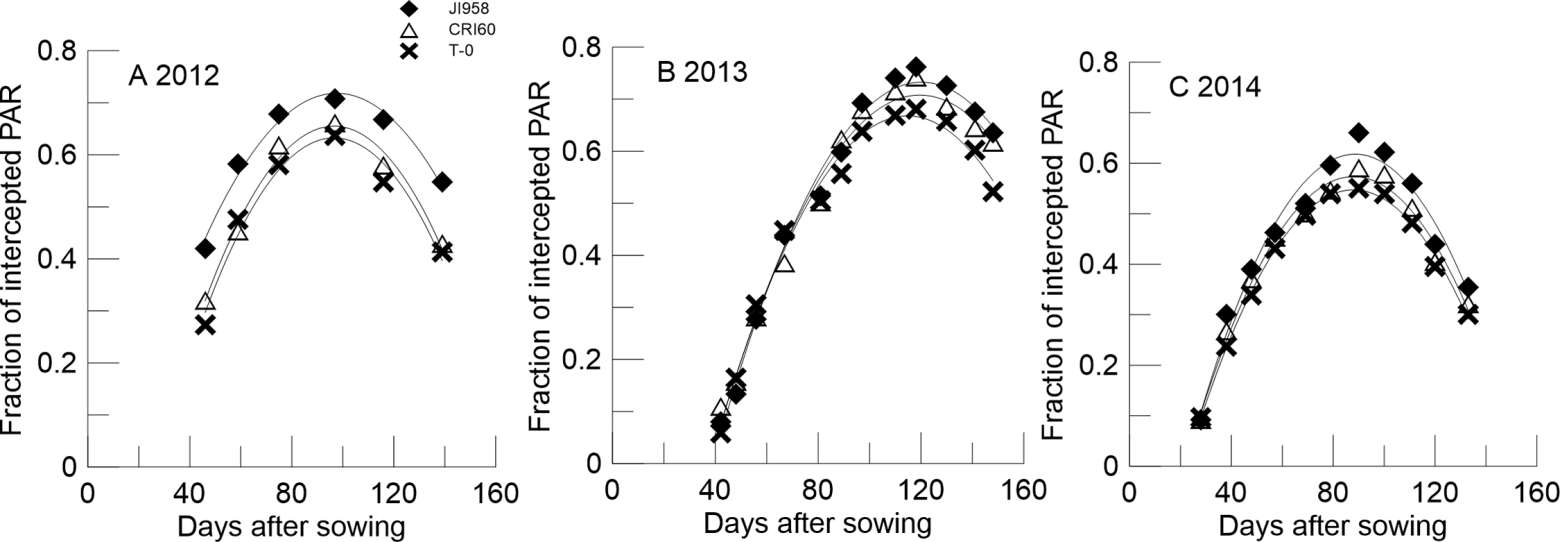
The variation of iPAR over the entire cotton growing period for Ji958, CRI60 and T-0 in 2012(A), 2013(B) and 2014(C).

A quadratic relationship of the days after sowing for the iPAR was observed during the cotton growth period (Table 2), and the determination coefficients were all above 0.91. Furthermore, the values of “a” were negative for iPAR simulation equations. The tendency of tPAR was completely antipodal. Three cultivars, Ji958, CRI60, and T-0, which were distinctly different in maturity and plant architecture, were chosen for detailed presentation regarding iPAR (Fig 1).

**Table 2:** iPAR simulation equations for ten cultivars in 2012, 2013 and 2014: Y = aX^2^+bX+c.

	Year 2012,N = 8		Year 2013,N = 12		Year 2014,N = 11	
	Equation	R^2^	Equation	R^2^	Equation	R^2^
CRI60	Y = -0.00013X^2^+0.02536X-0.56937	0.98	Y = -0.00011X^2^+0.02521X-0.79829	0.99	Y = -0.00013X^2^+0.02292X-0.43455	0.98
113	Y = -0.00015X^2^+0.02746X-0.60618	0.96	Y = -0.00013X^2^+0.02848X-0.91292	0.99	Y = -0.00014X^2^+0.02499X-0.51185	0.96
Ji228	Y = -0.00013X^2^+0.02688X-0.67895	0.97	Y = -0.00009X^2^+0.02343X-0.74890	0.99	Y = -0.00014X^2^+0.02518X-0.49954	0.98
Ji958	Y = -0.00010X^2^+0.02048X-0.28136	0.98	Y = -0.00011X^2^+0.02666X-0.86932	0.99	Y = -0.00014X^2^+0.02465X-0.47447	0.97
Lu28	Y = -0.00013X^2^+0.02536X-0.52721	0.99	Y = -0.00010X^2^+0.02327X-0.72443	0.98	Y = -0.00011X^2^+0.02091X-0.39578	0.97
915	Y = -0.00011X^2^+0.02219X-0.47481	0.98	Y = -0.00010X^2^+0.02398X-0.79142	0.98	Y = -0.00011X^2^+0.01911X-0.30333	0.93
CRI79	Y = -0.00013X^2^+0.02536X-0.54063	0.98	Y = -0.00009X^2^+0.02279X-0.71264	0.99	Y = -0.00010X^2^+0.01872X-0.29146	0.91
3799	Y = -0.00011X^2^+0.01981X-0.28272	0.96	Y = -0.00010X^2^+0.02371X-0.74363	0.99	Y = -0.00015X^2^+0.02609X-0.50582	0.98
T-0	Y = -0.00013X^2^+0.02533X-0.59050	0.96	Y = -0.00011X^2^+0.02578X-0.81081	0.99	Y = -0.00013X^2^+0.02214X-0.42546	0.99
6913			Y = -0.00010X^2^+0.02457X-0.82988	0.98	Y = -0.00012X^2^+0.02266X-0.45646	0.96

Y: Estimated value of iPAR; X: Days after sowing.

The characteristic tendency of iPAR variation throughout the whole growth period among the three cultivars was the same in all years. iPAR was highest for Ji958, followed by CRI60, and lowest for T-0. Specifically, in 2012, iPAR for Ji958 approximately ranged from 42.00–70.70% over the cotton development stage; for CRI60, it was 31.98–66.05%, and for T-0 it was 27.26–63.71%. The respective iPAR values were 7.98–76.22%, 10.98–74.19%, 5.90–68.11% in 2013 and 9.16–66.02%, 9.22–59.08%, 9.62–55.08% in 2014. However, during the decline stage in 2012, iPAR gradually decreased to 54.80%, 42.92%, and 41.35% for Ji958, CRI60 and T-0, respectively. In 2013, it decreased to 63.48%, 61.66%, 52.16%, and in 2014, to 35.37%, 32.15%, 30.07%, respectively.

Additionally, the estimated times when values reached their peak were 102, 98, 97 days after sowing for Ji958, CRI60, and T-0, respectively. The values peaked at 121, 117, 115 days after sowing in 2013 and at 88, 88, 85 days after sowing 2014, respectively. In all case, values peaked in the blossoming and boll-forming stages.

### 2. Spatial distribution of tPAR within the canopy of different cultivars

**Fig 2:**
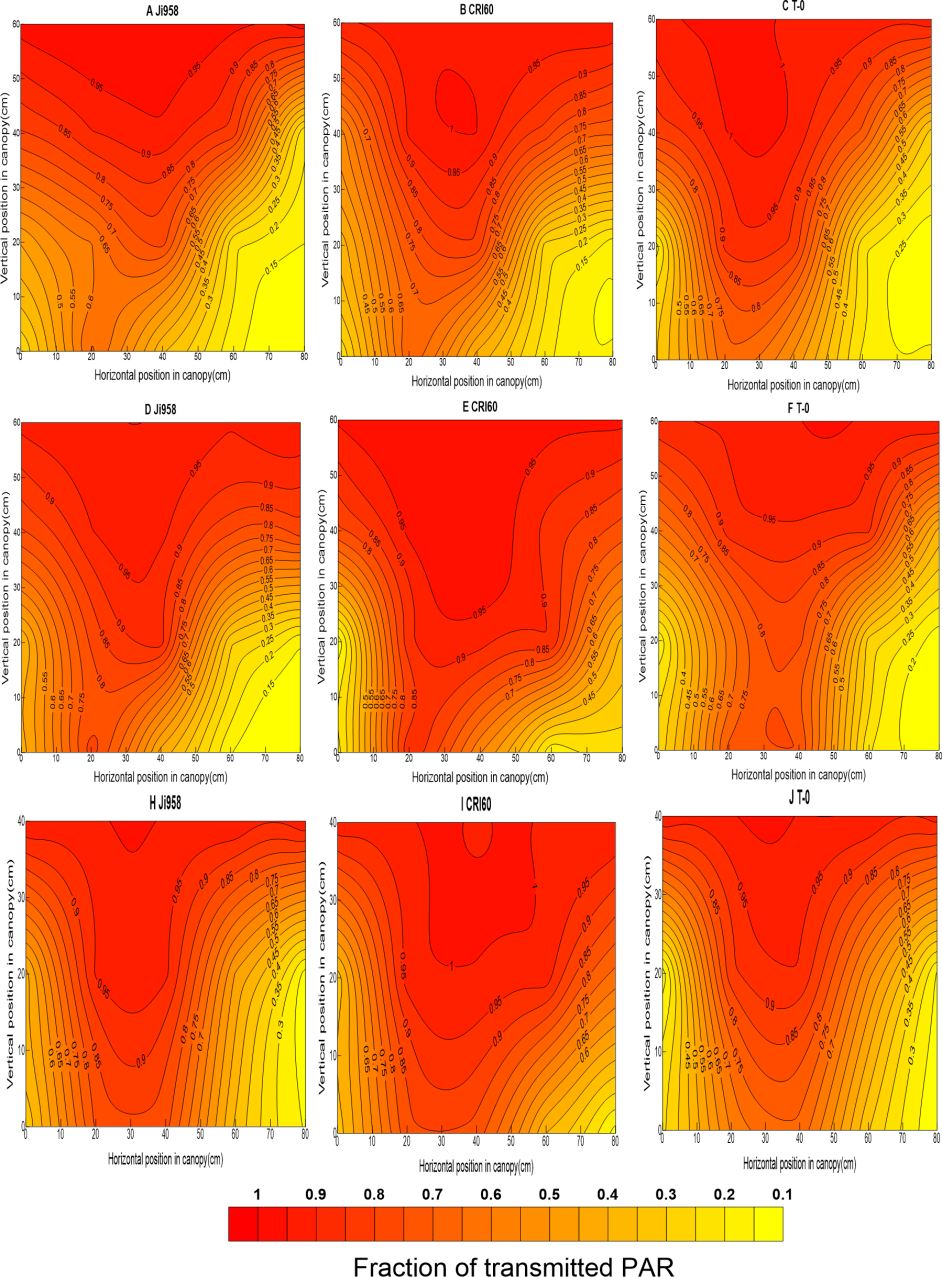
Vertical and horizontal distribution of tPAR at the squaring stage for Ji958, CRI60, T-0 in 2012 (A, B, C), 2013 (D, E, F) and 2014(H, I, J).

**Fig 3:**
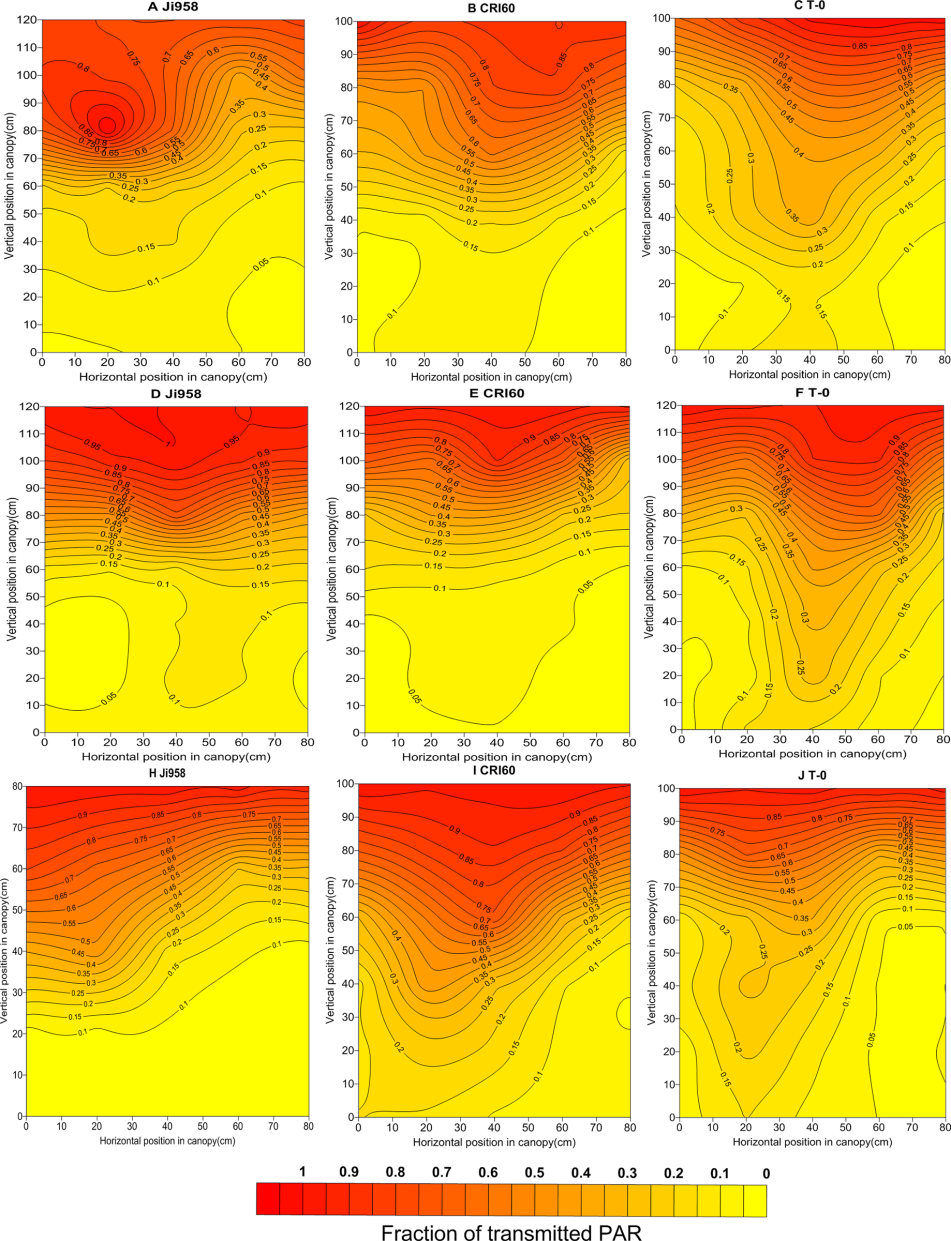
Vertical and horizontal distribution of tPAR at the blossming and boll forming stage for Ji958, CRI60, T-0 in 2012 (A, B, C), 2013 (D, E, F) and 2014(H, I, J).

Values of tPAR in other positions in the canopy were calculated by spatial interpolation with Surfer software V11 (Golden Software Inc., USA), and contour plots were drawn. These plots provided a straightforward display of the characteristics of light spatial distribution in the canopy. Here, we emphatically analyzed the results in two stages of cotton development: one measurement in the squaring stage, when the plants had not covered the inter-row, and another measurement in the blossoming and boll-forming stage, when the canopy had closed. As before, Ji958, CRI60, and T-0 are described in detail (Fig 2 and Fig 3).

According to our study, at the early growing stage, all of the contour plots behaved in a “V” shape. As an example, in 2012, from bottom to top within the canopy of Ji958, the estimated value of tPAR ranged from 0.44 to 0.98 in the 10 cm horizontal position and 0.12–0.95 in the 70 cm horizontal position near the cotton rows, whereas it was 0.35–0.95 in the 40 cm horizontal position in the mid-point of the rows. However, the peak value of tPAR appeared in the 20 cm position, where it ranged from 0.65 to 0.99. In canopies of CRI60 the values were 0.44–0.96 and 0.11–0.99 in the 10 cm and 70 cm vertical positions and 0.37–1.0 in the 40 cm horizontal position. AS before, the maximum value appeared near the 20 cm position and ranged from 0.68–1.0.In T-0 canopies, the values were 0.55–1.0, 0.25–0.96, and 0.57–0.99 in the 10 cm, 70 cm and 40 cm horizontal positions, respectively. Similarly, the most PAR was also transmitted through the canopy near the 20 cm position, with a value of 0.73–1.0.

At later growing stage, as branches stretch and the leaf area expands, the canopy begins to close, causing the spatial distribution of tPAR in the canopy to change obviously. For example, near the cotton rows (10 cm or 70 cm horizontal positions), the tPAR was approximately 0.04–1.0 (Ji958), 0.10–0.87 (CRI60), and 0.13–0.80 (T-0) from the bottom to the top of the canopy. Meanwhile, at the midpoint (40 cm horizontal position), it was approximately 0.05–1.0, 0.13–0.90, and 0.17–0.96 for Ji958, CRI60 and T-0, respectively. However, in the transverse direction, at the bottom of the canopy tPAR was mainly under 0.1, but in the 40 cm vertical position, it was approximately 0.13, 0.15, 0.17 near the rows and 0.15, 0.20, 0.30 at the midpoint between the rows, respectively. At the 80 cm vertical position, near the top of the canopy, tPAR was 0.5, 0.55 and 0.65 near the rows and 0.65, 0.75, 0.75 between rows, respectively.

### 3. LAI development and biomass accumulation of different cultivars

Throughout the entire growth period, LAI development presented a quadratic tendency with highly significant correlation coefficients between 0.91–0.97(Table 3). In the early developmental stage, LAI expanded with plant growth, and then a gradual reduction occurred due to the senescence of lower leaves after reaching a peak. The estimated maximum LAI was calculated using the fitting equation. Again using Ji958, CRI60 and T-0 as representatives, the maximum LAI values in 2012 were 2.9, 2.7 and 2.4, respectively; while it was 3.8, 3.2, 3.4 in 2013 and 2.7, 2.5, 2.3 in 2014. However, the times when LAI arrived the peaks were 106, 101 and 102 days after sowing in 2012, and 124, 115, 121 days in 2013, 97, 97, 94 days in 2014, respectively, showing that the development process was faster in 2014 than in 2012 and 2013.

**Table 3:** LAI simulation equations for ten cultivars in 2012, 2013 and 2014: Y = aX^2^+bX+c.

	Year 2012, N = 8		Year 2013, N = 12		Year 2014, N = 11	
	Equation	R^2^	Equation	R^2^	Equation	R^2^
CRI60	Y = -0.00062X2+0.12553X-3.67514	0.91	Y = -0.00064X2+0.14742X-5.33729	0.92	Y = -0.00058X2+0.11226X-2.91327	0.93
113	Y = -0.00058X2+0.11624X-3.36312	0.94	Y = -0.00062X2+0.14501X-5.24924	0.92	Y = -0.00068X2+0.12666X-3.28689	0.91
Ji228	Y = -0.00059X2+0.12928X-3.88643	0.91	Y = -0.00058X2+0.14273X-5.23096	0.94	Y = -0.00059X2+0.11740X-3.10270	0.92
Ji958	Y = -0.00059X2+0.12506X-3.74256	0.92	Y = -0.00058X2+0.14438X-5.22178	0.96	Y = -0.00061X2+0.11858X-3.10044	0.91
Lu28	Y = -0.00053X2+0.10935X-3.11201	0.91	Y = -0.00056X2+0.13682X-4.99868	0.93	Y = -0.00061X2+0.11703X-3.08269	0.91
915	Y = -0.00042X2+0.08419X-2.17705	0.93	Y = -0.00061X2+0.14445X-5.21465	0.91	Y = -0.00050X2+0.09733X-2.48724	0.93
CRI79	Y = -0.00066X2+0.13619X-4.01277	0.92	Y = -0.00052X2+0.12896X-4.73296	0.92	Y = -0.00051X2+0.09987X-2.53879	0.91
3799	Y = -0.00046X2+0.09565X-2.66149	0.97	Y = -0.00061X2+0.14461X-5.19996	0.94	Y = -0.00048X2+0.09448X-2.36633	0.95
T-0	Y = -0.00052X2+0.10567X-2.97648	0.93	Y = -0.00056X2+0.13598X-4.88478	0.95	Y = -0.00056X2+0.10544X-2.65401	0.93
6913			Y = -0.00055X2+0.13924X-5.13799	0.94	Y = -0.00055X2+0.10819X-2.79901	0.92

Y: Estimated value of LAI; X: Days after sowing.

The logistic model adequately described the total dry matter evolution, and the correlation coefficients were as high as 0.95–0.99 (Table 4). In the model, the value of K represented the theoretical maximum of dry matter accumulation that the crops can produce during the whole development period. Although in 2012, the K of Ji958 was slightly less than that of CRI60, the K value of Ji958 was always apparently higher than that of CRI60 in 2013 and 2014, and the value of T-0 was consistently lower. In addition, the onset time of rapid dry matter accumulation for Ji958 in 2012 was 70 days after sowing, but it was 72 and 68 days for CRI60 and T-0, respectively. In 2013, it was 94, 96and 83 days, respectively, and in 2014, 78, 72, 65 days. T-0 was always the earliest, but Ji958 and CRI60 failed to maintain a consistent variation tendency. Furthermore, the estimated values of the maximum rate of dry matter accumulation for Ji958, CRI60, and T-0 were 226.8, 219.2 and 185.8 in 2012; 255.9, 236.1, 226.1 in 2013; and 264.0, 216.3, 198.5 in 2014, respectively.

**Table 4:** Biomass accumulation simulating equations for ten cultivars in 2012, 2013 and 2014: Y = K/ (1+ae^bX^).

	Year 2012, N = 8		Year 2013, N = 12		Year 2014, N = 11	
	Equation	R^2^	Equation	R^2^	Equation	R^2^
CRI60	Y = 12910.40/(1+495.0783e-0.06791X)	0.99	Y = 17879.22/(1+599.7073e-0.05281X)	0.96	Y = 14303.40/(1+284.9703e-0.06048X)	0.98
113	Y = 12305.86/(1+338.26448e-0.06186X)	0.97	Y = 17333.46/(1+663.2541e-0.05416X)	0.95	Y = 13249.59/(1+495.8531e-0.07331X)	0.99
Ji228	Y = 12430.84/(1+598.3143e-0.07413X)	0.99	Y = 19253.77/(1+578.1132e-0.05281X)	0.97	Y = 14179.45/(1+403.1712e-0.06856X)	0.99
Ji958	Y = 12288.18/(1+640.00948e-0.07383X)	0.99	Y = 19695.94/(1+494.351e-0.05197X)	0.96	Y = 18561.322/(1+321.26993e-0.05689X)	0.98
Lu28	Y = 13587.70/(1+274.08778e-0.05653X)	0.98	Y = 19262.48/(1+607.8447e-0.05317X)	0.96	Y = 15904.46/(1+405.2435e-0.06414X)	0.99
915	Y = 11771.16/(1+197.88770e-0.05707X)	0.99	Y = 20033.59/(1+552.3341e-0.05193X)	0.97	Y = 13776.82/(1+283.6423e-0.06148X)	0.99
CRI79	Y = 12042.52/(1+100.58916e-0.04943X)	0.99	Y = 18498.56/(1+495.5449e-0.05137X)	0.95	Y = 17307.104/(1+246.64185e-0.05428X)	0.97
3799	Y = 10048.31/(1+294.70908e-0.06803X)	0.99	Y = 18711.76/(1+523.5059e-0.05219X)	0.96	Y = 17541.675/(1+239.84087e-0.05424X)	0.97
T-0	Y = 10975.58/(1+389.12715e-0.06773X)	0.98	Y = 13861.05/(1+852.6834e-0.06524X)	0.99	Y = 11665.28/(1+314.3358e-0.06805X)	0.99
6913			Y = 19521.00/(1+659.0740e-0.05383X)	0.98	Y = 14976.13/(1+434.2733e-0.06801X)	0.99

Y: Estimated value of biomass accumulation; X: Days after sowing.

### 4. Relationship between iPAR and LAI in each year

**Fig 4:**
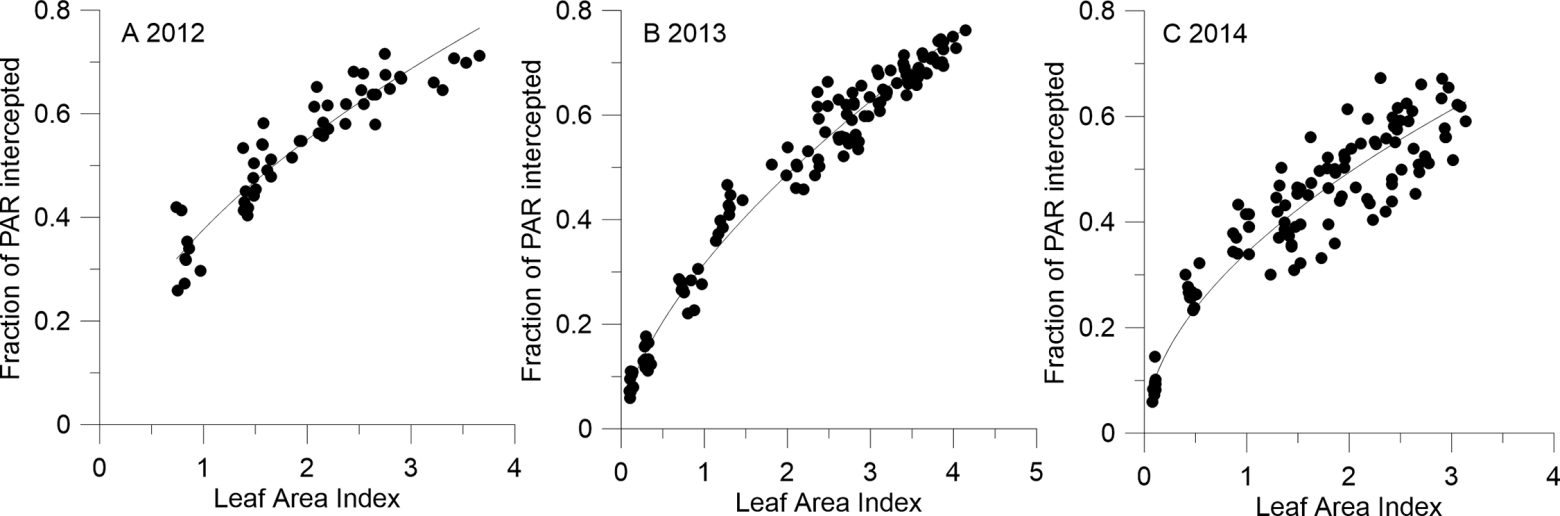
Relationship between LAI and iPAR in 2012 (A), 2013 (B) and 2014(C).

iPAR showed a highly significant exponential correlation with LAI of all genotypes in each year according to our experiment (R^2^≥0.86, P>|t|:<0.001)(Fig 4). The fitted equations were as follows:

$$\text{Equation A}\;(2012):\;\text{ln}(Y)=0.55\times \text{ln}(X)-0.98,N=54,R^{2}=0.86;$$

$$\text{Equation B}\;(2013):\;\text{ln}(Y)=0.62\times \text{ln}(X)-1.15,N=120,R^{2}=0.98;$$

$$\text{Equation C}\;(2014):\;\text{ln}(Y)=0.53\times \text{ln}(X)-1.07,N=110,R^{2}=0.92.$$

In each equation, X represents LAI, and Y represents iPAR.

### 5. Relationship between accumulated PAR interception and biomass accumulation for different cultivars

**Fig 5:**
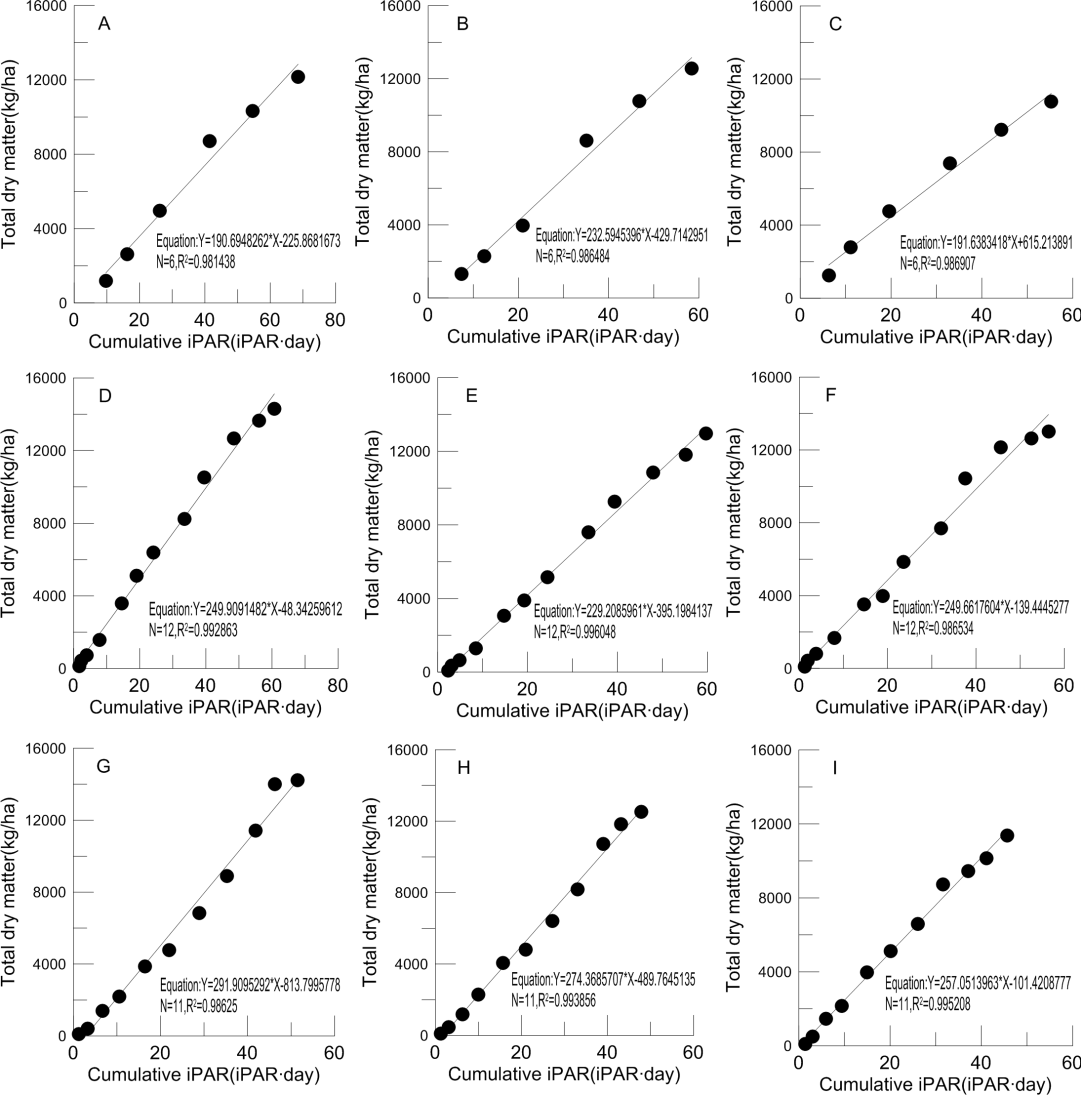
Relationship between the cumulative iPAR and total dry matter accumulation during the whole growing period for Ji958, CRI60 and T-0 in 2012(A, B, C), 2013(D, E, F) and 2014(G, H, I)

Linear regression models were applied to the relationship between cumulative biomass and intercepted PAR accumulation to estimate RUE (Fig 5), and the slopes give a specific value of the increment by intercepting more units of PAR [22], which can evaluate the productivity of the crop.

According to our results, the values for Ji958, CRI60 and T-0 were 190.69, 232.59 and 191.64 in 2012, while the values was 249.91, 229.21, 249.66 in 2013 and 291.91, 274.37, 257.05 in 2014, respectively. Unfortunately, the results did not show a consistent pattern. The higher value in 2014 may be attributable to more suitable weather for cotton growth.

## Discussion

Light interception by plant canopy plays an important role that determines plant biomass production and growth [27]. Therefore, numerous previous researchers have focused on improving the light micro-environment in the canopy to increase the potential crop productivity [28–29]. In particular, the effects of plant architecture on light distribution and interception within the canopy are studied frequently [30].

However, previous measurement methods of light distribution either ignored the mutual interaction between plant organs [31] or required substantial labor and capital costs or expensive equipment [32]. In the present study, an accurate and convenient investigation of light distribution in the heterogeneous and irregular canopy was conducted using a geo-statistical sampling method. Afterwards, the detailed characteristics were estimated using spatial interpolation and accumulated tPAR was calculated using Simpson 3/8 rules.

In our study, iPAR varied as a mountain-like curve over the whole growth period. It increased rapidly during plant development and LAI expansion in the early stage and then declined as foliage senesced. This was in agreement with the results of Li [24] and Xue [33]. Furthermore, an exponential correlation between iPAR and LAI was fitted, similar to findings of L.A. Vargas, G.A. Maddonni, R.A. Ruiz [5, 6, 34].

The iPAR and LAI values of cultivars like Ji958 were clear higher than those of the other two types throughout the entire period. This is due to the different growth habits among the varieties. In the vegetative growth stage, cultivars like Ji958 had more exuberant development, which is beneficial to produce more shoots and leaves and build a wide canopy quickly. This was conducive to intercepting more radiation and growing quickly. Consequently, it went into a virtuous cycle for biomass production. What is more, more green leaves resulted in deferred senescence for the plant and sustained LAI and iPAR at high levels for longer. The date when Ji958 reached peak values of iPAR and LAI was later than CRI60 and T-0, further supporting this point.

Moreover, according to our results, light distribution within the cotton canopy presented a significant heterogeneous variance, which strongly agreed with the findings of ZHI [18] and Leuchner [35]. At the early developmental stage, prior to canopy closure, radiation transmission decreased with depth from the top to the bottom of the canopy. Furthermore, it decreased more quickly and abundantly near rows than at-between-row positions. This is due to the more numerous interstices in positions close to the middle [11, 35]. However, the maximum of tPAR occurred closer to the west rather than in the center between two rows, which should be attributed to the low solar altitude in the morning.

At the late stage of cotton development, light distribution was significantly different in the fully developed canopies of the cultivars with different plant types. PAR in canopies of cultivars like Ji958, with an incompact structure, decreased most rapidly in the upper layers. This resulted from exuberant vegetative organs. Overlong branches and superfluous leaves led to a dense canopy, especially in the upper positions. The closed upper canopy prevented radiation from being transmitted into the lower layers, which contributed to most of the absorption of radiation being focused at the top of the canopy. This result was consistent with Gretchen’s report, which indicated that the rapid attenuation of PAR mainly occurred in the upper canopy of cotton genotypes that had larger and fairly flat leaves, compared with genotypes which had more erect leaves [36]. However, cultivars like T-0, with short fruiting branches and compact nodes, failed to build a closed canopy because of a lack of vegetative organs. That resulted in a high light loss ratio because more radiation was transmitted through or across the canopy instead of being absorbed by the leaves. Meanwhile, in cultivars like CRI60, the light penetration within the canopy was more gradual and homogeneous, benefiting from the moderate branch length and foliage size.

These results strongly demonstrate that plant type and canopy structure significantly influence light distribution and interception in the canopy, which has been reported by many previous researchers, including Zhang, Vos J, Ruth Kaggwa-Asiimwe, Stewart, and Wiechers, *et al* [13–14,22,37–38].

A significant positive linear relationship was found between the cumulative amount of iPAR and biomass accumulation, which was consistent with many previous studies [5, 39–41]. The cultivars like Ji958 intercepted total radiation more than other cultivars during the whole growth period, and they produced more biomass. Although these cultivars were planted in essentially the same environment and received similar field management, we did not find a specific change in RUE as we did for iPAR. This should be interpreted as RUE being strongly affected by not only environmental conditions and management factors but also crop genotype and photosynthetic characteristics. This result was also suggested by Sinclair et al. [42] and Stöckle et al. [43]. Furthermore, many predecessors also expressed that a more uniform distribution of radiation in the canopy by increased PAR penetration to lower layers can also enhance the photosynthetic efficiency of the crop population [44–46]. Therefore, further research on the photosynthetic characteristics of these diverse genotypes would be necessary to find the cause of differences in RUE.

M.P. Bange suggested that early-maturing cotton cultivars possessed a priority to partition dry matter to the reproductive organs [47], which restrained the development of vegetative growth. On the one hand, this decreased light interception; on the other hand, it decreased the duration of green leaf area. Both of these factors adversely affected cumulativ light interception. Moreover, Quisenberry and Roark also found that there were some evidence suggested a trade-off between early maturation and yield potential in cotton [48].

Overall, cultivars with a heavy canopy and long growth period were able to intercept more solar radiation and produce more biomass. This result was consistent with the findings of Li, who found that higher LAI and longer growth duration produced higher fresh forage yield in an experiment evaluating and screening varieties of manure used for growing peas [49], and it also agreed with Tharakan and his co-workers, who indicated that a higher LAI and long canopy duration were essential for gaining more biomass production [50]. Furthermore, in our study more biomass accumulation was better explained by a greater amount of PAR interception than by higher RUE.

## Conclusion

In conclusion, the results from the present study indicate the following:(i) light distribution in the canopy had a significant spatial heterogeneity and was greatly influenced by canopy structures; (ii) cumulative PAR interception was highly and positively associated with green leaf area and duration; (iii) biomass accumulation was correlated positively and linearly with the strength of radiation interception; (iv) a closed and serried canopy would contribute to intercepting more solar radiation, but it was not necessarily beneficial for the improvement of RUE.

These findings will help growers develop new cultivation strategies aimed at improving light interception affected by canopy architecture, such as a more appropriate plant density or row spacing for diverse cultivars. Moreover, it can also assist breeding researchers in the selection of genotypes with an optimal canopy architecture and appropriate maturity, to improve the light environment within the canopy and thus the photosynthetic capability.

However, as an economic crop, lint yield is the ultimate goal for cotton planting. Regrettably, research on yield traits is lacking in this article. A study on how to partition more biomass to fruit than to vegetative organs is imperatively requested.

## References

[1] Hall AE (2001). Crop response to environment, Chapter 4, crop physiological responses to light, photosynthesis, and respiration, p.40.

[2] Lambers H, Chapin FS III, L Pons TL (2008). Plant Physiological Ecology (Second Edition), Chapter 2.3 response of photosynthesis, p.26-27.

[3] Gosse G, Varlet-Granche C, Bonhomme R, Chattier M, Allirand J, Lemaire G (1986). "Maximum dry matter production and solar radiation intercepted by a canopy." Agronomie 6(1): 47–56.

[4] Louarn G, Lecoeur J, Lebon E (2008). "A three-dimensional statistical reconstruction model of grapevine (Vitis vinifera) simulating canopy structure variability within and between cultivar/training system pairs." Ann Bot 101(8): 1167–1184. 10.1093/aob/mcm170 18202006 PMC2710267

[5] Vargas LA, Andersen MN, Jensen CR, Jorgensen U (2002). "Estimation of leaf area index, light interception and biomass accumulation of Miscanthus sinensis 'Goliath' from radiation measurements." Biomass & Bioenergy 22(1): 1–14.

[6] Maddonni GA, Otegui ME (1996). "Leaf area, light interception, and crop development in maize." Field Crops Research 48(1): 81–87.

[7] Reinhardt D, Kuhlemeier C (2002). "Plant architecture." Embo Reports 3(9): 846–851. 12223466 10.1093/embo-reports/kvf177 PMC1084230

[8] Barthelemy D, Caraglio Y (2007). "Plant architecture: A dynamic, multilevel and comprehensive approach to plant form, structure and ontogeny." Annals of Botany 99(3): 375–407. 17218346 10.1093/aob/mcl260 PMC2802949

[9] Russell G, et al (1989). Absorption of radiation by canopies and stand growth In: Rusell G, Marshall B, Jarvis P, editors. Plant canopies: their growth, form and function. Cambridge, UK: Cambridge University Press, 1989. p. 21–41.

[10] Sinoquet H, Thanisawanyangkura S, Mabrouk H, Kasemsap P (1998). "Characterization of the light environment in canopies using 3D digitising and image processing." Annals of Botany 82(2): 203–212.

[11] Sarlikioti V, de Visser PHB, Marcelis LFM (2011). "Exploring the spatial distribution of light interception and photosynthesis of canopies by means of a functional-structural plant model." Ann Bot 107(5): 875–883. 10.1093/aob/mcr006 21355008 PMC3077986

[12] Larsen DR, Kershaw JA (1996). "Influence of canopy structure assumptions on predictions from Beer's law. A comparison of deterministic and stochastic simulations." Agricultural and Forest Meteorology 81(1–2): 61–77.

[13] Zhang WY, Tang L, Yang X, Liu LL, Cao WX, Zhu Y (2015). "A simulation model for predicting canopy structure and light distribution in wheat." European Journal of Agronomy 67: 1–11.

[14] Vos J, Evers JB, Buck-Sorlin GH, Andrieu B, Chelle M, de Visser PHB (2010). "Functional-structural plant modelling: a new versatile tool in crop science." Journal of Experimental Botany 61(8): 2101–2115. 10.1093/jxb/erp345 19995824

[15] Rey H, Dauzat J, Chenu K, Barczi JF, Dosio GAA, Lecoeur J (2008). "Using a 3-D virtual sunflower to simulate light capture at organ, plant and plot levels: contribution of organ interception, impact of heliotropism and analysis of genotypic differences." Ann Bot 101(8): 1139–1151. 10.1093/aob/mcm300 18218705 PMC2710280

[16] Ma Y, Wen MP, Li BG, Wang XP, Guo Y (2007). "Efficient model for computing the distribution of direct solar radiation in maize canopy at organ level." Transactions of the Chinese Society of Agricultural Engineering 23(10): 151–155.

[17] Kiniry J, Johnson MV, Mitchell R, Vogel K, Kaiser J, Bruckerhoff S, et al (2011). "Switchgrass Leaf Area Index and Light Extinction Coefficients." Agronomy Journal 103(1): 119–122.

[18] Zhi XY, Han YC, Mao SC, Wang GP, Feng L, Yang BF, et al (2014). "Light Spatial Distribution in the Canopy and Crop Development in Cotton." PLoS One 9(11). 10.1371/journal.pone.0113409 PMC4237451 25409026

[19] Melo JD, Carreno EM, Calvino A, Padilha-Feltrin A (2014). "Determining spatial resolution in spatial load forecasting using a grid-based model." Electric Power Systems Research 111: 177–184.

[20] Institute of Cotton Research of CAAS. (2013). Cotton cultivation in China Shanghai Scientific and Technical Publisher, 3 2013.p.123–124.

[21] Jost P, Whitaker J, Brown SM, Bednarz C (2006). Use of plant growth regulators as a management tool in cotton Univ. of Georgia Bulletin, p.1305.

[22] Kaggwa-Asiimwe R, Andrade-Sanchez P, Wang GY (2013). "Plant architecture influences growth and yield response of upland cotton to population density." Field Crops Research 145: 52–59.

[23] Tang L, Zhu XC, Cao MY, Cao WX, Zhu Y (2012). "Relationships of rice canopy PAR interception and light use efficiency to grain yield." The Journal of Applied Ecology 23(5): 1269–1276 22919837

[24] Li YB, Mao SC, Feng L (2012). “Spatial distribution characteristics of photosynthetic active radiation in cotton canopy based on geo-statistics”. Transactions of the Chinese Society of Agricultural Engineering (Transactions of the CSAE), 28(22): 200–206. (In Chinese with English abstract).

[25] O'Neal ME, Landis DA (2002). "An inexpensive, accurate method for measuring leaf area and defoliation through digital image analysis." Journal of Economic Entomology 95(6): 1190–1194. 12539831 10.1603/0022-0493-95.6.1190

[26] Xue XP, Guo WQ, Wang YL, Zhang LJ, Zhou ZG (2006). "Characteristics of dynamic increase of cotton biomass at different N levels." Cotton Science 18(6): 323–326.

[27] Escobar-Gutierrez AJ, Combes D, Rakocevic M, de Berranger C, Eprinchard-Ciesla A, Sinoquet H, et al (2009). "Functional relationships to estimate Morphogenetically Active Radiation (MAR) from PAR and solar broadband irradiance measurements: The case of a sorghum crop." Agricultural and Forest Meteorology 149(8): 1244–1253.

[28] Fila G, Sartorato I (2011). "Using Leaf Mass per Area as predictor of light interception and absorption in crop/weed monoculture or mixed stands." Agricultural and Forest Meteorology 151(5): 575–584.

[29] Gonias ED, Oosterhuis DM, Bibi AC (2011). "Light interception and radiation use efficiency of okra and normal leaf cotton isolines." Environmental and Experimental Botany 72(2): 217–222.

[30] Watanabe T, Hanan JS, Room PM, Hasegawa T, Nakagawa H, Takahashi W, et al (2005). Rice morphogenesis and plant architecture: measurement, specification and the reconstruction of structural development by 3D architectural modelling. Ann. Bot. 95, 1131–1143. 15820987 10.1093/aob/mci136 PMC4246908

[31] Andrieu B, Ivanov N, Boissard P (1995). "SIMULATION OF LIGHT INTERCEPTION FROM A MAIZE CANOPY MODEL CONSTRUCTED BY STEREO PLOTTING." Agricultural and Forest Meteorology 75(1–3): 103–119.

[32] Tournebize R, Sinoquet H (1995). "LIGHT INTERCEPTION AND PARTITIONING IN A SHRUB/GRASS MIXTURE." Agricultural and Forest Meteorology 72(3–4): 277–294.

[33] Xue HY, Han YC, Li YB, Mao SC, Wang GP, Feng L, et al (2015). “Spatial distribution of light interception by different plant Population densities and its relationship with yield”. Field Crops Research 184: 17–27.

[34] Ruiz RA, Bertero HD (2008). "Light interception and radiation use efficiency in temperate quinoa (Chenopodium quinoa Willd.) cultivars." European Journal of Agronomy 29(2–3): 144–152.

[35] Leuchner M, Hertel C, Menzel A (2011). "Spatial variability of photosynthetically active radiation in European beech and Norway spruce." Agricultural and Forest Meteorology 151(9): 1226–1232

[36] Sassenrath-Cole GF (1995). “Dependence of canopy light distribution on leaf and canopy structure for two cotton (*Gossypium*) species”. Agricultural and Forest Meteorology 77: 55–72.

[37] Stewart DW, Costa C, Dwyer LM, Smith DL, Hamilton RI, Ma BL (2003). "Canopy structure, light interception, and photosynthesis in maize." Agronomy Journal 95(6): 1465–1474.

[38] Wiechers D, Kahlen K, Stutzel H (2011). "Evaluation of a radiosity based light model for greenhouse cucumber canopies." Agricultural and Forest Meteorology 151(7): 906–915.

[39] Friday JB, Fownes JH (2001). "A simulation model for hedgerow light interception and growth." Agricultural and Forest Meteorology 108(1): 29–43.

[40] Tesfaye K, Walker S, Tsubo M (2006). "Radiation interception and radiation use efficiency of three grain legumes under water deficit conditions in a semi-arid environment." European Journal of Agronomy 25(1): 60–70.

[41] Singer JW, Meek DW, Sauer TJ, Prueger JH, Hatfield JL (2011). "Variability of light interception and radiation use efficiency in maize and soybean." Field Crops Research 121(1): 147–152.

[42] Sinclair TR, Muchow RC (1999). "Radiation use efficiency." Advances in Agronomy, Vol 65 65: 215–265.

[43] Stöckle C, Kemanian A (2009). Crop radiation capture and use efficiency: a framework for crop growth analysis In: Sadras V.O., Calderini D.F. (Eds.), Crop Physiology: Applications for Genetic Improvement and Agronomy. Academic Press, San Diego, CA, USA, pp. 145–170.

[44] Kuroiwa S (1970). Total photosynthesis of a foliage in relation to inclination of leaves In: Setllk I. (Editor), Prediction and Measurement of Photosynthetic Productivity. Centre for Agricultural Publishing and Documentation, Wageningen, pp. 79–89.

[45] Aikman DP (1989). Potential increase in photosynthetic efficiency from the redistribution of solar radiation in a crop. J. Exp. Bot., 40: 855–864.

[46] Herbert TJ (1991). Variation in interception of the direct solar beam by top canopy layers. Ecology, 72:17–22.

[47] Bange MP, Milroy SP (2000). "Timing of crop maturity in cotton—Impact of dry matter production and partitioning." Field Crops Research 68(2): 143–155.

[48] Quisenberry JE, Roark B (1976). Influence of indeterminate growth habit on yield and irrigation water-use efficiency in upland cotton. Crop Sci. 16: 762–765.

[49] Li ZH (2011). “Initial Evaluation of Pea Germplasm for Forage and Green Manure in Ecotone”. Acta Agriculturae Boreali-Sinica 26(4):67–71.

[50] Tharakan PJ, Volk TA, Nowak CA, Ofezu GJ (2008). "Assessment of Canopy Structure, Light Interception, and Light-use Efficiency of First Year Regrowth of Shrub Willow (Salix sp.)." Bioenergy Research 1(3–4): 229–238.

